# A quality of life questionnaire for patients with scapula alata (SA-Q): development and validation

**DOI:** 10.1186/s12891-020-03284-4

**Published:** 2020-04-21

**Authors:** Sigrid Tibaek, Janne Gadsboell

**Affiliations:** Department of Occupational Therapy and Physiotherapy, Rigshospitalet Glostrup, Copenhagen University Hospital, Valdemar Hansens Vej 13, DK-2600 Glostrup, Denmark

**Keywords:** Content validity, Scapula alata, QoL questionnaire

## Abstract

**Background:**

No quality of life (QoL) questionnaire exists for patients with scapula alata (SA). The objective of this study was to develop and validate a QoL questionnaire for SA patients.

**Methods:**

A team consisting of experts (*n* = 7) and SA patients (n = 7) developed, through five continuous phases, a QoL questionnaire for SA patients (SA-Q). The developed questionnaire consists of 21 items, grouped in five domains: physical symptoms (five items), work (four items), sport and leisure activities (four items), life style (four items) and emotions (four items).

Content and face validity for the SA-Q questionnaire were evaluated by a sample of 48 (90%) out of 53 SA patients recruited from a university hospital. The Content Validity Index (CVI) and modified kappa index (κ*) assessed the relevance of SA-Q questionnaire.

**Results:**

The SA patients evaluated 20 (95%) out of 21 items as excellent for content validity (I-CVI > 0.78, κ* > 0.74), one (5%) item was considered as good (I-CVI < 0.78, 0.60 < κ* < 0.74). The average scale (S-CVI/ave) for the entire SA-Q questionnaire was 0.93 indicating an excellent content validity.

**Conclusions:**

This study presents the development and validation of content validity of the first QoL questionnaire for SA patients. The SA-Q questionnaire has potential clinical implications for detected changes concerning the different items during rehabilitation.

**Clinical trials:**

Not relevant.

## Background

Scapula alata (SA) [1–3] is a clinical condition present in both children [4, 5] and adults [6]. The condition is characterised by sudden shoulder pain of typically a few weeks duration followed by rapid fatigue of the arm, reduced muscle strength and inability to elevate the affected arm above shoulder level [6]. Consequently, patients experience various limitations in work, sport, leisure and life style, leading to a major impact on their quality of life (QoL) [6].

Multiple pathologies can lead to SA. Palsy of the serratus anterior muscle caused by injury to the long thoracic nerve is considered the most common cause [7]. Injury to the nerve can be caused by infection [8], traction or compression of the nerve [9] or damage to the nerve associated with surgery or accident [10, 11]. The serratus anterior muscle is essential for the scapula motion and stability; palsy of the muscle causes malalignment and winging of the scapula resulting in severe biomechanical alterations of scapula and the shoulder complex in general. Consequently, the patient is not able to elevate the arm above shoulder level on the affected side.

Various treatments have been recommended [10, 12]. However, there are no treatment programmes to guide clinicians. Recently we presented in detail a rehabilitation programme [6] and evaluated its impact using a patient-reported measurement tool the Western Ontario Rotator Cuff Index (WORC). However, test for content validity of the WORC in patients with SA indicated that only approximately half of the items in WORC were relevant for SA patients [13]. Subsequently, a further literature review still showed no QoL measurement tool for SA patients. The objective of this study was to develop and validate a QoL questionnaire targeted at patients with SA.

## Methods

### Questionnaire development

The development of the QoL questionnaire for SA patients was carried out by a SA team and was done in the Danish language.

### The SA team

The SA Team consisted of experts (*n* = 7) and SA patients (n = 7). The experts were recruited from: a specific shoulder-clinic, Skulderklinikken Viborg Aps (*n* = 1) and three Danish university hospitals, Viborg Regional Hospital (*n* = 2); Aalborg University Hospital (*n* = 3) and Rigshospitalet Glostrup (n = 1) from January 1st to March 31th, 2018.

The SA patients were recruited from the Department of Occupational Therapy and Physiotherapy, Rigshospitalet Glostrup, Copenhagen University Hospital from June 3th to October 17th, 2018.

The inclusion criteria for the experts were: a) medical or physiotherapy education and b) clinical experience in treatment of SA patients for at least five years. Inclusion criteria for the SA patients were: a) outpatients diagnosed with SA caused by injury to the long thoracic nerve, b) current or former (< 3 years) patients who were receiving or had undergone an SA rehabilitation programme [6] and c) aged 15 years or older. Exclusion criteria were: a) history of shoulder injury, b) additional neurological disorders and c) bone abnormality.

### Procedure

The experts were invited by an e-mail. Having accepted, they received by post a cover letter including: a) information on background and aim of the study, b) practical information (meeting place and frequency) and c) a form for them to summarise their demographic and medical education characteristics.

When the experts had agreed to participate, they were asked to fill out the characteristic form and return it by mail to the research leader within two weeks. In cases of no reply, reminders were sent after two weeks and, if necessary, after five weeks.

The SA patients were successively invited verbally and after accepting they received a written cover letter from the Department of Occupational Therapy and Physiotherapy, Rigshospitalet Glostrup. The cover letter included: a) background and aim of the study, b) information and instructions, c) informed consent and d) a pre-paid envelope. When the SA patients had agreed to participate, they were asked to return the signed informed consent to the author by pre-paid post within two weeks. In cases of no reply reminders were sent after two weeks and, again if necessary, after five weeks.

### Process

The development process was guided by the methods described in the US Food and Drug Administration (FDA) Guidance for Industry: Patient-reported Outcome Measures [14, 15].

**Fig. 1:**
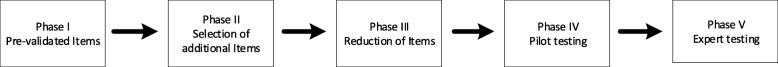
Flow chart presenting the development of the QoL Questionnaire for Patients with scapula alata (SA-Q)

The process was generated in five (I-V) continuous phases [16, 17] (Fig. 1).

### Phase I: pre-validated items

Recently, an expert panel (*n* = 6) in the SA field and a sample of SA patients (*n* = 49) attempted to validate the WORC Index as an applicable QoL questionnaire for SA patients [18]. The results indicated acceptable content validity of 10 (48%) out of 21 items. The accepted items were grouped in the following domains: physical symptoms (1 out of 6 items), sport/recreation (3 out of 4 items), work (4 out of 4 items), life style (0 out of 4 items) and emotions (2 out of 3 items).

In the recent content validity study, the SA experts and patients suggested that relevant items regarding physical activities and working aspects were missing, while items regarding life style were too gender-specific. These aspects are important for the development of a QoL questionnaire for SA patients.

### Phase II: selection of additional items

In accordance with the aim of developing a QoL questionnaire targeted at SA patients, the experts and patients in the team were asked to identify and select as many additional QoL items as possible corresponding to the following domains: a) physical symptoms and activities, b) work and c) non-gender specific life style aspects which were previously found to be poorly covered.

The additional items were picked up based on patients’ and experts’ experience - the latter being inspired by other shoulder-specific QoL questionnaires e.g. Disabilities of the Arm, Shoulder and Hand (DASH) questionnaire [19].

In total 120 items were suggested, 85 (71%) items by the experts and 35 (29%) items by the patients. Seventy items, including the 10 pre-validated items, turned out to be duplicates, leaving 50 different items for development of the future QoL questionnaire.

### Phase III: reduction of items

The experts performed an evaluation of the 50 items through standard item reduction analysis, using a 5-point Likert scale (2 = definite yes to − 2 definite no) for each item. Items with mean for all experts score < 0, were removed [20].

This reduced the number of items to 40. The experts discussed the relevance of these 40 items for the SA patients. Each item was evaluated by a 4-level Likert scale (not relevant = 0; seldom relevant = 1; sometimes relevant = 2 and always relevant = 3). The cut-off point for acceptable items was a rating of 2 or 3.

Finally, a first draft of the QoL questionnaire for SA patients was developed consisting of 21 items.

### Phase IV: pilot test

This first draft of the QoL questionnaire with 21 items was used through an individual face-to-face interview, the Three- Step Test Interview [21]. The interviews were conducted by the co-author and SA patients (*n* = 10) who followed the inclusion and exclusion criteria as described above. The aim was to obtain responses regarding relevance and understanding of the items.

Based on this pilot testing of the first draft of a QoL questionnaire the second draft was developed.

### Phase V: experts testing

The SA experts discussed and evaluated the second draft of the QoL questionnaire and needed three rounds to reach consensus for which items should be included in the final version of a QoL questionnaire for SA patients.

The final version was entitled “A Quality of Life Questionnaire for patients with scapula alata (SA-Q)” and consisted of 21 items grouped in five domains: physical symptoms (five items), work (four items), sport and leisure activities (four items), life style (four items) and emotions (four items) (Table 1).

**Table 1:**
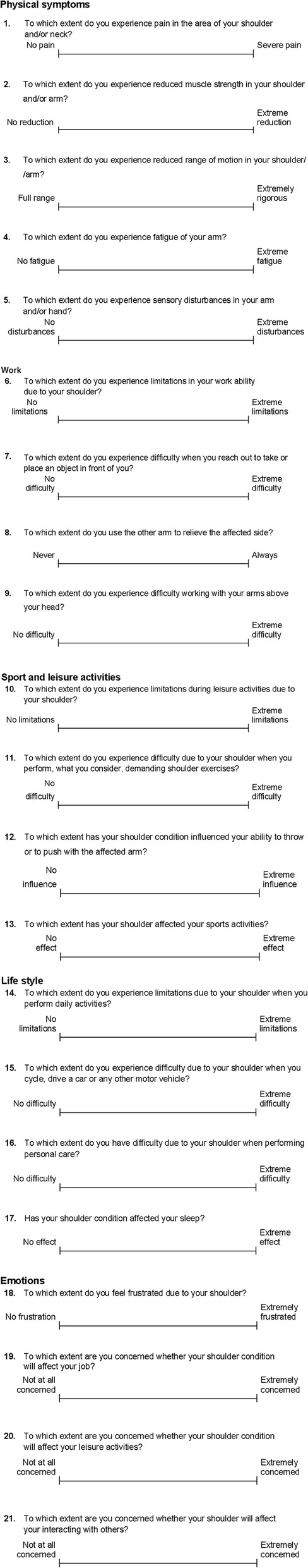
A Quality of Life Questionnaire for Patients with scapula alata (SA-Q)

In the instructions for completing the SA-Q questionnaire the patients were advised to score each item on a visual analogue scale (VAS) based on their experiences in the previous two weeks. A score of 0 indicates no impact of symptom on QoL, while a score of 100 is worst case. The scores were summed up with a possible range of 0 to 2100.

For presenting the results in a more clinically meaningful format it is recommended that the score is reported as a QoL index by subtracting the total score from 2100, dividing by 2100 and multiplying by 100, that is the formula is: SA-Q index = 100 * $\frac{2100- total\ score}{2100.}$

The scale of SA-Q index will now go from 0% (major impact on QoL) to 100% (no impact on QoL).

The SA patients were informed that this SA-Q questionnaire would takes an average of 10–15 min to complete.

The SA-Q questionnaire was developed in Danish as the original version. For use of SA-Q in worldwide cultures, the psychometrics need to be tested for validity in the speaking language of the country [22].

## Translation into English

The SA-Q questionnaire was translated to English according to the guidelines by Guillemin 1993 [22] and Beaton 2000 [23] which include

- translation of the questionnaire by two independent translators; b) synthesis of translations, in which the two versions were compared; c) back translation by two further translators and d) committee review by a professional committee consisting of a physiotherapist, an occupational therapist and the two researchers.
  The translated SA-Q questionnaire has not been tested yet by interview in a sample of native English-speaking SA patients.

### Questionnaire validation

According to the criteria of the COSMIN Standards (COnsensus-based Standards for the selection of health status Measurement INstruments) [24], the domain validity includes three measurement properties: content validity, construct validity, and criterion validity.

The content validity includes face and content validity.

### Participants

A sample of 48 (90%) participated from the 53 SA patients recruited for face and content validity of the developed SA-Q questionnaire for SA patients. The patients were recruited from the outpatient clinic at the Departments of Occupational Therapy and Physiotherapy, Rigshospitalet Glostrup from January 1st to September 2nd, 2019. The inclusion and exclusion criteria for the patients were the same as described above in the SA team paragraph.

The SA patients were invited verbally and by a written cover letter. The cover letter included: a) background and aims of the study, b) information and instructions, c) informed consent and d) a pre-paid envelope. If the SA patients agreed to participate, they were asked to return the signed informed consent to the research leader by post within two weeks. In cases of no reply reminders were sent after two weeks and again after five weeks.

The participants were asked to evaluate the relevance of the SA-Q questionnaire for SA patients The participants received a cover letter plus: a) information and instructions, b) two evaluation forms: one for the individual items and one for the total SA-Q questionnaire, c) a sheet for qualitative comments in understanding and using the SA-Q questionnaire and e) a prepaid envelope. The participants were asked to return the sheets within two weeks. In cases of no reply reminders were sent after two weeks and five weeks.

### Face validity

The term face validity is defined by the COSMIN panel as “the degree to which a measurement instrument looks as though it is an adequate reflection of the construct to be measured” [24]. It is a subjective assessment and therefore there are no standards regarding how it should be assessed and quantified [25].

In term of face validity, the floor and ceiling effect of the SA-Q questionnaire were measured in the group of SA patients currently undergoing the rehabilitation programme [6].

Floor effects were considered to be present if ≥15% scored an item as 0 (lowest possible score) and ceiling effects were considered to be present if ≥15% scored an item as 100 (VAS scale 0–100) (highest possible score) on the SA-Q questionnaire [26].

### Content validity

The term content validity is defined as “The degree to which an instrument has the appropriate sample of items of the construct being measured” [27].

The content validity of SA-Q questionnaire was evaluated by the Content Validity Index (CVI) and a modified kappa index (κ*). The evaluation in terms of relevance of the SA-Q questionnaire was assessed by the Content Validity Index (CVI) [28], which include:

*1.* Evaluation of each item in the SA-Q questionnaire; in terms of relevance for SA patients this was measured by Items-Content Validity Index (I-CVI).

2. Evaluation of the entire SA-Q questionnaire; in term of relevance for SA patients this was measured by the Scale-Content Validity Index (S-CVI).

The evaluation was rated as a number on a 4-point ordinal scale (1 = not relevant, 2 = somewhat relevant, 3 = quite relevant, 4 = highly relevant.

### Data analysis

The point for acceptable value for content value for each item is a rating of 3 or 4.

I-CVI was calculated as the part of SA patients rating an item either 3 or 4 *and presented in decimal in the Result section according to Polit* et al.*(27)*. The S-CVI were calculated as the average of the I-CVIs for all items on the scale (S-CV I/Ave) rating the entire questionnaire by 3 or 4.

Any I-CVI rated higher than or equal to 0.78 by 6 or more SA patients is considered excellent. Subsequently Polit et al. recommend 0.78 as excellent regardless of the number of experts. The recommendation for S-CVI/Ave is 0.90 or higher [2, 29].

To address the limitations of CVI, each I-CVI was adjusted for chance agreement by calculating the modified kappa statistic (κ*) [29, 30]. To compute the modified kappa, the probability of chance agreement is computed first: P_c_ = $\frac{N!}{A!\left(N-A\right)!}$ * 0,5 ^N^ where N is the number of SA patients and A is the number of agreements of good relevance (rating 3 and 4). Then the κ* was calculated for each item using the formula κ* = $\frac{I- CVI- pc}{1-p\mathrm{c}}$ [28].

According to the standards of Fleiss and Cicchetti & Sparrow the value of each κ* was evaluated as: poor (κ* < 0.40), fair (0.40 < κ* < 0.59), good (0.60 < κ* < 0.74) or excellent (κ* > 0.74) [31, 32].

In this study, the SA patients were asked to evaluate the entire SA-Q questionnaire overall as a QoL measurement for SA patients using the same four-point scale defined as S-CVI.

The SA patients were asked to quantify by written feedback content validity of SA-Q questionnaire using the CVI supplied. The CVI is an index of inter-rater agreement. There are alternative methods to CVI as described and discussed by Polit et al. [29], however it seems that the CVI has been preferred in health-related fields as an indicator of content validity.

Statistical analysis was carried out using Microsoft Excel Office 2013. Median and interquartile range (IQR) are presented for the small sample and mean and standard deviation (SD) for the larger sample.

The level of statistical significance was set to *p* < 0.05.

## Results

### Participants

The experts (*n* = 7) in the SA team were females (100%), educated physiotherapists (100%), medium 48 (IQR 42–55) years and medium 12 (IQR 11–18) years clinical experiences in treatment of SA patients. The demographic and medical characteristics of patients in the SA team (n = 7), pilot test (*n* = 10) and validation test (*n* = 48) are presented in Table 2.

**Table 2:** The demographic and medical characteristics of patients participating in the scapula alata team, pilot test and validation test

Characteristics	SA team (*n* = 7) No. (%)	Pilot test (*n* = 10) No. (%)	Validation test (*n* = 48) No. (%)
Age, years^a^	44 (43–48)	50 (37–52)	43 (36–54)
Gender			
Female	3 (43)	2 (20)	19 (40)
Male	4 (47)	8 (80)	29 (60)
Educational level			
Short education	1 (14)	2 (20)	3 (6)
Medium education	1 (14)	1 (10)	18 (37)
Academic education	5 (72)	7 (70)	27 (57)
Employment status			
Employed	7 (100)	7 (70)	38 (79)
Self-employed		2 (20)	2 (4)
Unemployed		1 (10)	1 (2)
Studying			5 (10)
Sick leave			1 (2)
Retired			1 (2)
Rehabilitation status			
Current	7 (100)	10 (100)	24 (50)
Former			24 (50)
Pathology			
N. Thoracicus affection	7 (100)	10 (100)	48 (100)
N. Accessorius affection			
Ethicology			
Trauma			
Mono neuritis	6 (86)	10 (100)	48 (100)
Surgery sequala			
Stretch/overload			
Infection sequala			
unknown	1 (14)		
Affected side			
Right	6 (86)	10 (100)	48 (100)
Left	1 (14)		
Dominant hand			
Right	7 (100)	8 (80)	41 (85)
Left		2 (20)	7 (15)

^a^Median and interquartile range (IQR)

### Validation

The evaluations of the newly developed SA-Q questionnaire were completed by all 48 participants.

### Content validity

The study indicated acceptable content validity for the SA-Q questionnaire. As presented in Table 3, 20 (95%) out of 21 items were evaluated as excellent content validity (I-CVI > 0.78, k* > 0.74), one (5%) item was evaluated as good (I-CVI < 0.78, 0.60 < κ* < 0.74). For the entire SA-Q questionnaire S-CVI/Ave = 0.93, indicating excellent content validity and for the five domains following: physical symptoms = 0.91, work = 0.95, sport and leisure activities = 0.94, life style = 0.88 and emotions = 0.85. All SA participant rated the total SA-Q questionnaire as a relevant QoL measurement for SA patients, resulting in S-CVI/_total_ = 1.00.

**Table 3:** Content validity of A Quality of Life Questionnaire for patients with scapula alata evaluated by 48 patients

		I-CVI	κ^*^	Evaluation
**Physical symptoms**				
**1.**	To which extent do you experience pain the area of your shoulder and/or neck?	0.94	0.94	excellent
**2.**	To which extent do you experience reduced muscle strength in your shoulder and/or arm?	1.00	1.00	Excellent
**3.**	To which extent do you experience reduced range of motion in your shoulder/arm?	0.94	0.92	Excellent
**4.**	To which extent do you experience fatigue of your arm?	0.90	0.90	Excellent
**5.**	To which extent do you experience sensory disturbances in your arm and/or hand?	0.79	0.79	Excellent
**Work**				
**6.**	To which extent do you experience limitations in your work ability due to your shoulder?	0.96	0.96	Excellent
**7.**	To which extent do you experience difficulty when you reach out to take or place an object in front of you?	0.96	0.96	Excellent
**8.**	To which extent do you use the other arm to relieve the affected side?	0.91	0.92	Excellent
**9.**	To which extent do you experience difficulty working with your arms above your head?	0.98	0.98	Excellent
**Sport and leisure activities**				
**10.**	To which extent do you experience limitations during leisure activities due to your shoulder?	0.98	0.96	Excellent
**11.**	To which extent do you experience difficulty due to your shoulder when you perform, what you consider demanding shoulder exercises?	0.94	0.92	Excellent
**12.**	To which extent has your shoulder condition influenced your ability to throw or to push with the affected arm?	0.92	0.92	Excellent
**13.**	To which extent has your shoulder affected your sports activities?	0.92	0.98	Excellent
**Life style**				
**14.**	To which extent do you experience limitations due to your shoulder when you perform daily activities?	0.92	0.90	Excellent
**15.**	To which extent do you experience difficulty due to your shoulder when you cycle, drive a car or another motor vehicle?	0.85	0.85	Excellent
**16.**	To which extent do you have difficulty due to your shoulder when performing personal care?	0.83	0.81	Excellent
**17.**	Has your shoulder condition affected your sleep?	0.92	0.90	Excellent
**Emotions**				
**18.**	To which extent do you feel frustrated due to your shoulder?	0.98	0.98	Excellent
**19.**	To which extent are you concerned whether your shoulder condition will affect your job?	0.89	0.90	Excellent
**20.**	To which extent are you concerned whether your shoulder condition will affect your leisure activities	0.94	0.94	Excellent
**21.**	To which extent are you concerned whether your shoulder will affect your interacting with others?	0.69	0.69	Good

κ^*^ Modified kappa index

### Face validity

Forty-three participants completed all 21 (100%) items in the SA-Q customaries, two participants completed 20 (96%) items, one seven (33%), while the SA-Q questionnaires were missing from two former SA patients. The values rated on the SA-Q questionnaire by the participants (*n* = 24) who currently receive the rehabilitation programme indicated no floor (6%) or ceiling (2%) effect.

### Qualitative comments

Qualitative comments were added by 34 participants as e.g. a) one participant refers to item 1 “I did not feel real pain, but more nuisance in the shoulder and neck”; b) one participant asks for a different word from “demanding” in item 11; c) one participant did not like the VAS scale for rating; d) while most participants (*n* = 11) welcomed the questionnaire as “a relevant and meaningful measure of QoL during the rehabilitation and as follow-up” and e) the reminding participants described in detail their difficulties in various physical function.

## Discussion

In this manuscript we present the development and validation of content validity of the first QoL questionnaire for SA patients. The developed SA-Q questionnaire consist of 21 items grouped in five domains: physical symptoms, work, sport and leisure activities, life style and emotions and the SA-Q is questionnaire is translated into English according to guidelines.

The validity in terms of content validity indicated acceptable content. In terms of face validity, the SA-Q questionnaire seems well understood, meaningful and without floor or ceiling effects.

### Previous studies

Recently, Vastamäki et al. reported an 11-items questionnaire for patients with serratus palsy, the Helsinki Serratus Palsy Index (HSP) [33]. The HSP Index was based on the results from content and construct validity analysis of the 21 items in the WORC Index [34] plus 4 additional items. However, the authors did not appropriately follow the guiding for development a patient-reported outcome measurement [14, 15, 25] and the patients who in 2017 rated the validity were diagnosed with serratus palsy between 1981 and 2008.

Moreover, the authors pointed out that there is a need for a QoL tool to measure level of symptoms and effect of treatment in patients with serratus palsy [33].

We have found no other studies to compare with this one.

### Questionnaire development

In phase III of the questionnaire development, a larger number of duplicate items were suggested by the experts and SA patients, which improves the item reduction process. However, there were arguments concerning some items e.g. in item 1 “To which extend extent do you experience pain ….? ”, The relevance of this item depends on the duration from the onset of the SA condition. Most patients experience severe neuropathic pain [35] at the onset. Subsequently, some SA patients experience nociceptive pain [35] or physical nuisance due to the scapular misalignment.

Another argument concerned specification versus generalisation of the items e.g. in item 14 “To which extent do you experience … … when you perform daily activities?”. Some experts preferred to cover a specific aspect of daily activities such as “when you are gardening?”, but this aspect was irrelevant for SA patients living in apartments. Another example concerning specification versus generalisation appeared in item 15 “To which extend … ..do you experience difficulty when you cycle, drive a car or ….? ” Particularly young SA patients living in a city use a cycle while SA patients living in the countryside generally drive cars.

For item 21 “To which … ...whether your shoulder will affect your interacting with others? ”, SA patients might not know exactly what is meant by the question (I-CVI = 0.69).

In phase II, developing of the SA-Q questionnaire, it was suggested and argued for including close social contact questions such as playing with or lifting up children, dancing or having sex, but excluded due to risk of low response rate to such questions, particularly concerning sexual activities [36].

### Methodological considerations

Several methodological issues need to be considered.

First, the lack of testing of all three types of validity. According to the COSMIN Standards [37] this questionnaire has to be tested also for reliability, construct validity and structural validity before it can be applied in clinical practice.

Second, the sample size of SA patients. For the validation the participants were successively recruited over eight months and the former SA patients over three years. It was not possible to estimate a correctly sample size of SA patients as far as we do not have any prevalence studies.

The representativeness of SA patients is also relevant.

Although children were not included in this study, the age of the participants ranged from 24 to 72 years in the SA patients. In term of gender most SA patients were male which is in line with the participants in the study by Vastamäki 2018 et al. [33]. There is no national SA database describing the characteristics of SA patients.

Third, the results of S-CVI/A indicated lower impact on QoL in life style and emotion domains. As pointed out previously, some items seem to confuse some participants. A larger number of items in the life style and emotion domains might probably have reduced the problem. However, when taking all of the other excellent items into account, the lower results of life style and emotion domains does not alter the fact that SA-Q is a content valid questionnaire for SA patients.

Finally, concerning the scapula alata affected side. All participants (100%) in the present study were affected on the right side of whom (84%, Table 2) were predominantly right-handed; this is in line with 89% of the participants in the study by Vastamäki et al. [33]. We need an explanation for this fact.

### Perspectives

This SA-Q questionnaire has high potential clinical implications detecting changes in the different items during rehabilitation. However, a test-retest reliability test is needed and the translated SA-Q questionnaire must be tested by interview in a sample of native English-speaking SA patients.

Later translation of SA-Q questionnaire to other languages followed by objective measurements are needed to support and heighten a documented effect of rehabilitation.

In addition, a national SA database is needed due to getting a step deeper in the ethology and pathology of the SA condition.

## Conclusions

The present study presents the development and validation of content validity of the first QoL questionnaire for SA patients.

The SA-Q questionnaire has been translated to English and for the future, it may have high potential clinical implications detecting changes concerning the different items during rehabilitation.

## Data Availability

The datasets used and analysed during the current study are available from the corresponding author on reasonable request.

## Abbreviations

ADA: The US Food and Drug Administration
CVI: Content Validity Index
DASH: Disabilities of the, Arm, Shoulder and Hand
I-CVI: Items-Content Validity Index
IQR: Inter quartile range
SA: Scapula alata
SA-Q: Quality of Life Questionnaire for patients with scapula alata
S-CVI: Scale_Content Validity Index
SD: Standard deviation
QoL: Quality of Life
WORC: Western Ontario Rotator Cuff Index

## References

[1] Vanderstraten J (2010). Scapula alata. La revue de la Médicine Générale.

[2] Waltz CFS, Strickland OL, Lenz ER (2016). Measurement in Nursing and Health research.

[3] Warner JJ, Navarro RA (1998). Serratus anterior dysfunction. Recognition and treatment. Clin Orthop Relat Res.

[4] Fjeldborg PK, Hansen TB (2012). Atypical cause of scapular winging due to exostosis of the scapula. Ugeskr Laeger.

[5] Scott DA, Alexander JR (2010). Relapsing and remitting scapular winging in a pediatric patient. Am J Phys Med Rehabil.

[6] Tibaek S, Gadsboell J (2015). Scapula alata: description of a physical therapy program and its effectiveness measured by a shoulder-specific quality-of-life measurement. J Shoulder Elb Surg.

[7] Vastamaki M, Kauppila LI (1993). Etiologic factors in isolated paralysis of the serratus anterior muscle: a report of 197 cases. J Shoulder Elb Surg.

[8] Bot SD, Terwee CB, van der Windt DA, Bouter LM, Dekker J, de Vet HC (2004). Clinimetric evaluation of shoulder disability questionnaires: a systematic review of the literature. Ann Rheum Dis.

[9] Safran MR (2004). Nerve injury about the shoulder in athletes, part 2: long thoracic nerve, spinal accessory nerve, burners/stingers, thoracic outlet syndrome. Am J Sports Med.

[10] Adriaenssens N, De Ridder M, Lievens P, Van Parijs H, Vanhoeij M, Miedema G (2012). Scapula alata in early breast cancer patients enrolled in a randomized clinical trial of post-surgery short-course image-guided radiotherapy. World J Surg Oncol.

[11] Connor PM, Yamaguchi K, Manifold SG, Pollock RG, Flatow EL, Bigliani LU (1997). Split pectoralis major transfer for serratus anterior palsy. Clin Orthop Relat Res.

[12] Ginn KA, Cohen ML (2005). Exercise therapy for shoulder pain aimed at restoring neuromuscular control: a randomized comparative clinical trial. J Rehabil Med.

[13] Gadsboell J, Tibaek S (2017). Validity of a shoulder-specific quality of life questionnaire, the Western Ontario rotator cuff index, for patients with scapula alata. JSES Open Access.

[14] Santosh P, Lievesley K, Fiori F, Singh J (2017). Development of the tailored Rett intervention and assessment longitudinal (TRIAL) database and the Rett evaluation of symptoms and treatments (REST) questionnaire. BMJ Open.

[15] FDA (2009). Patient-reported Outcome Measures: Use in medical product development to support labelling claims.

[16] Willert CB, Holmich LR, Thorborg K (2015). Developing and validating of patient-reported questionnaires - part 1. Ugeskr Laeger.

[17] Willert CB, Holmich LR, Thorborg K (2015). Developing and validating of patient-reported questionnaires - part 2. Ugeskr Laeger.

[18] Gadsboell J, Tibaek S (2017). Validity of a shoulder-specific quality of life questionaire, the Western Ontario rotator cuff index, for patients with scapula alata. J Shoulder Elb Surg.

[19] Kirkley A, Griffin S, Dainty K (2003). Scoring systems for the functional assessment of the shoulder. Arthroscopy..

[20] Hudak PL, Amadio PC, Bombardier C (1996). Development of an upper extremity outcome measure: the DASH (disabilities of the arm, shoulder and hand) [corrected]. The upper extremity collaborative group (UECG). Am J Ind Med.

[21] Hak T, van der Veer K, Jansen H (2008). The three-step test-interview (TSTI): an observation-based method for pretesting self-completion questionnaires. Survey Research Methods.

[22] Guillemin F, Bombardier C, Beaton D (1993). Cross-cultural adaptation of health-related quality of life measures: literature review and proposed guidelines. J Clin Epidemiol.

[23] Beaton DE, Bombardier C, Guillemin F, Ferraz MB (2000). Guidelines for the process of cross-cultural adaptation of self-report measures. Spine (Phila Pa 1976).

[24] Mokkink LB, Terwee CB, Patrick DL, Alonso J, Stratford PW, Knol DL (2010). The COSMIN study reached international consensus on taxonomy, terminology, and definitions of measurement properties for health-related patient-reported outcomes. J Clin Epidemiol.

[25] de Vet HCW, Terwee CB, Mokkink LB, Knol DL (2017). Measurement in medicine.

[26] Josefsson KA, Ekdahl C, Jakobsson U, Gard G (2013). Swedish version of the multi dimensional health assessment questionnaire -- translation and psychometric evaluation. BMC Musculoskelet Disord.

[27] Polit DF, Beck CT (2006). The content validity index: are you sure you know what's being reported?Critique and recommendations. Res Nurs Health.

[28] Lynn MR (1986). Determination and quantification of content validity. Nurs Res.

[29] Polit DF, Beck CT, Owen SV (2007). Is the CVI an acceptable indicator of content validity? Appraisal and recommendations. Res Nurs Health.

[30] Wynd CA, Schmidt B, Schaefer MA (2003). Two quantitative approaches for estimating content validity. West J Nurs Res.

[31] Cicchetti DV, Sparrow SA (1981). Developing criteria for establishing interrater reliability of specific items: applications to assessment of adaptive behavior. Am J Ment Defic.

[32] Fleiss LJ, Levin B, Paik MC. Statistical Methods for Rates and Proportions. 3rd Edition ed. Hoboken: Wiley; 2003.

[33] Vastamaki M, Ristolainen L, Vastamaki H, Laimi K, Saltychev M (2018). Validity and internal consistency of the Helsinki Serratus palsy index for patients with serratus palsy. J Shoulder Elb Surg.

[34] de Witte PB, Henseler JF, Nagels J, Vliet Vlieland TP, Nelissen RG (2012). The Western Ontario rotator cuff index in rotator cuff disease patients: a comprehensive reliability and responsiveness validation study. Am J Sports Med.

[35] Ellison DL (2017). Physiology of pain. Crit Care Nurs Clin North Am.

[36] Prah P, Johnson AM, Nardone A, Clifton S, Mindell JS, Copas AJ (2015). Asking about sex in general health surveys: comparing the methods and findings of the 2010 health survey for England with those of the third National Survey of sexual attitudes and lifestyles. PLoS One.

[37] Mokkink LB, de Vet HCW, Prinsen CAC, Patrick DL, Alonso J, Bouter LM (2018). COSMIN risk of Bias checklist for systematic reviews of patient-reported outcome measures. Qual Life Res.

